# Inflammation, infection and depression: an evolutionary perspective

**DOI:** 10.1017/ehs.2019.15

**Published:** 2019-12-09

**Authors:** Caroline Doyle, Walker A. Swain, Holly A. Swain Ewald, Paul W. Ewald

**Affiliations:** 1 Department of Biology, Bellarmine University, Louisville, KY 40205, USA; 2 Department of Lifelong Education, Administration, and Policy, University of Georgia, Athens, GA 30602, USA; 3 Department of Biological Sciences, University of Louisville, Louisville, KY 40292, USA

**Keywords:** Inflammation, depression, sexually transmitted infection, *Chlamydia trachomatis*, tryptophan

## Abstract

The evolutionary basis for clinical depression is not well understood. A growing body of literature that is not based on evolutionary logic links inflammation to depression. Integration of these findings with an evolutionary framework for depression, however, needs to address the reasons why the body's inflammatory response would be regulated so poorly that it would result in incapacitating depression. Pathogen induction of inflammation offers an explanation, but the extent to which the association between inflammation and depression can be attributed to general inflammation as opposed to particular effects of pro-inflammatory pathogens remains unclear. This paper reports a study of sexually transmitted pathogens, which addresses this issue. Although several sexually transmitted pathogens were associated with depression according to bivariate tests, only *Chlamydia trachomatis* and *Trichomonas vaginalis* were significantly associated with depression by a multivariate analysis that accounted for correlations among the pathogens. This finding is consistent with the hypothesis that infection may contribute to depression through induction of tryptophan restriction, and a consequent depletion of serotonin. It reinforces the idea that some depression may be caused by specific pathogens in specific evolutionary arms races with their human host.

## Graphical abstract

**Figure:**
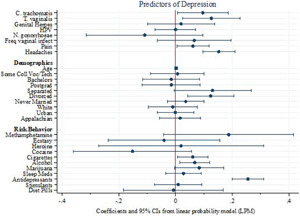

**Media summary:** Infection-associated depression correlates with *Chlamydia trachomatis* more strongly than general inflammatory responses.

## Introduction

The evolutionary reasons for the widespread presence of clinical depression are unclear. One line of evolutionary reasoning suggests that depression may cause individuals to shift away from unattainable goals (Nesse 2019). This explanation, however, seems inadequate for prolonged, incapacitating depression which should be purged by natural selection (Coyne 2010; Nesse 2019). The adaptive argument for short-term depression, however, may help explain the presence of adaptive neurological circuitry which then could function counter-productively when depression is prolonged and intractable. One hypothesis within this category of explanations invokes mismatches between modern and ancestral environments. Modern environments may have situational traps, such as economic immobility, which could keep depressed individuals from changing their living circumstances and thereby rebound from low mood (Nesse 2019). An alternative mismatch hypothesis proposes that the neocortex is particularly vulnerable to damaging environmental agents and that clinical depression results from the elevated presence of such hazards in modern relative to ancestral environments (Galecki and Talarowska 2017).

An alternative category of explanation invokes infection. Depression has been linked to innate immunological responses to infection and therefore could be interpreted as part of a mechanism that may facilitate recovery, for example, by encouraging rest, or may prevent additional infections in the depressed individual or kin (Anders *et al.*, 2013; Kinney and Tanaka 2009; Nesse 2019; Raison and Miller 2013b). As is the case with adaptive explanations of short-term depression that rely on goal-shifting, these hypotheses of infection-induced depression do not account well for prolonged, incapacitating depression. It is presumed that the fitness costs of prolonged, incapacitating depression must be incurred in order to obtain the even greater protective benefits of depression.

Clinical depression is distinguished from temporarily suppressed mood largely on the basis of its persistence for at least two weeks (American Psychiatric Association 2013). Accordingly, low mood associated with acute infectious disease is generally considered to be a manifestation of the disease rather than clinical depression. Persistent infections, however, might contribute to prolonged and debilitating depression that has not yet been attributed to infection. Evolutionary considerations emphasize that manifestations of infectious disease could benefit the host, parasite or neither (Ewald 1980). The reasoning leading to this conclusion is based on the coevolutionary instability that is associated with the evolutionary conflicts of interest between parasite and host. In this context, persistent, debilitating depression is not presumed to be an adaptation of the host or the infectious agent. Rather, an adaptive basis for depression may be manifested with an increased intensity or persistence that is beneficial to neither host nor pathogen but results instead from a disruption of the normal regulation of depression, a side effect of the coevolutionary arms race between particular parasites and their hosts.

Recent research has focused on the possibility that inflammation may play a causal role. Depression has been correlated with levels of inflammatory proteins (Khandaker *et al.* 2014; Pasco *et al.* 2010a, 2010b) and autoimmunity (Benros *et al.* 2013), but anti-inflammatory treatment has ameliorated depression inconsistently across studies (Baune 2017; Cubala and Landowski 2014; Eyre *et al.* 2015; O. Kohler *et al.* 2014, 2016a) and among patients within studies that have different levels of inflammation (Raison *et al*. 2013).

Infections have been suggested as instigators of immunological responses that could lead to depression (Benros *et al.* 2013; Canli 2014; Doyle *et al.* 2015; Maes *et al.* 2009; Miller *et al.* 2009; Raison and Miller 2013b). Several pathogens that cause persistent infections have been associated with depression. Human papillomavirus (HPV) has been associated with depressive symptoms in human immunodeficiency virus (HIV)-infected patients (Dodd *et al.* 2009; Lopez *et al.* 2013). *Chlamydia trachomatis* has been associated with depression in women tested during their annual check-ups (Doyle *et al.* 2015). A meta-analysis (Wang *et al.* 2014) found significant associations of depression with several persistent pathogens but did not account for correlations among these pathogens.

Although associations between infection and depression are often interpreted as occurring through the intervening variable of inflammation (Benros *et al.* 2013; Miller *et al.* 2009; Raison and Miller 2013b), several findings emphasize the need to consider more specific effects of infectious agents. In a low- to middle-income population in Detroit, cytomegalovirus was correlated with depression, but inflammatory markers were not (Simanek *et al.* 2014). Similarly, cytomegalovirus was correlated with depression in elderly Latinos in northern California, but inflammatory markers and several other persistent pathogens (herpes simplex virus, varicella zoster virus, *Helicobacter pylori* and *Toxoplasma gondii*) were not (Simanek *et al.* 2018b). In a nationwide US study cytomegalovirus and *H. pylori* were associated with depression in women, but a marker of inflammation (C-reactive protein) was not (Simanek *et al.* 2018a). In the meta-analysis mentioned above (Wang *et al.* 2014), an association of CMV with depression fell just short of statistical significance, but this analysis did not include any of the more recent studies by Simanek and colleagues cited above.

The variation in the association of depression with both inflammation and infection is consistent with the looseness of the associations of depression with indicators of inflammation and the caveat that depression should not be categorized simply as an inflammatory disorder (Raison and Miller 2013a). Associations of depression with specific infectious agents are difficult to interpret because different infections may occur in the same patient, particularly if they are transmitted by the same route. Sexual transmission, in particular, may favor co-occurrence because sexually transmitted infections are often inapparent and nearly always persistent. The contribution of a sexually transmitted pathogen to depression may therefore be inconspicuous because of asymptomatic infection and ambiguous because it could co-occur with other sexually transmitted pathogens that do not contribute to depression.

Inflammatory responses are complex and variable. Joint consideration of infection and inflammation raises the possibility that some pathogens may affect aspects of immunological responses in ways that lead to depression, whereas others may not. It was hypothesized (Doyle *et al.* 2015) that the association of depression with *C. trachomatis* resulted from persistent restriction of tryptophan, which the body invokes in association with inflammation as an adaptation to defend against infection (Olive and Sassetti 2016; Schmidt and Schultze 2014). This restriction may be chronically stimulated by *C. trachomatis* because this bacterium synthesizes its own tryptophan (Aiyar *et al.* 2014) and thus may survive in the presence of tryptophan restriction. Tryptophan is the precursor of serotonin, which plays a role in modulating mood (Fakhoury 2016; S. Kohler *et al.* 2016b; Kupfer *et al.* 2012); restricted tryptophan levels may therefore result in chronically depleted serotonin and hence low mood (Akers and Tan 2006).

The study by Doyle *et al.* (2015), which correlated *Chlamydia trachomatis* with depression, did not find significant associations with the other sexually transmitted pathogens that were evaluated: *Neisseria gonorrhoeae*, *Treponema pallidum*, *Candida albicans*, *Trichomonas vaginalis*, HPV and HIV. The study, however, was based on only 500 women, and sample sizes for *C. trachomatis* were greater than for most of the other tested pathogens (Doyle *et al.* 2015). A larger study population is needed to determine whether sexually transmitted pathogens other than *C. trachomatis* are associated with depression. One particularly relevant uncertainty pertains to *T. vaginalis*; it was associated with a doubling of the risk of depression, but this numerical association was not statistically significant (Doyle *et al.* 2015). Resolution of these uncertainties bears on whether efforts to prevent infection-induced depression should focus on particular pathogens or inflammation in general.

To address this issue we conducted a study using data from the Kentucky Women's Health Registry at the University of Kentucky's Center for the Advancement of Women's Health, which includes health-related information from over 17,000 women. We focus on women to build on the previous study of women (Doyle *et al.* 2015) and because effects of infection on depression might be stronger in women than in men as a result of the reliance on tryptophan restriction as part of elevated innate immunity during the luteal phase of the menstrual cycle (Hrboticky 1989; Doyle 2015). At that time tryptophan restriction can occur in response to estrogen (through effects on dendritic cells and macrophages; Xiao *et al.* 2004) even though other aspects of immune function are suppressed.

## Methods

Reproductive-aged women 18–40 years of age were selected as subjects from the Kentucky Women's Health Registry (currently ‘Wellness, Health and You’). Participants were recruited from throughout Kentucky using brochures at the University of Kentucky health care clinics, state and county health departments, county agricultural extension offices, offices of private physicians, women's professional organizations, homemaker organizations and health-related events. Participants completed a registry questionnaire electronically or on paper after providing consent. All information was self-reported.

Although depression can be manifested by a variety of indicators, a duration of at least two weeks is a generally agreed upon threshold for distinguishing clinical depression from transient depression (https://www.psychiatry.org/patients-families/depression/what-is-depression). We therefore included in the depressed category anyone who responded that they had been ‘down, depressed or hopeless’ for a duration of at least two weeks at some point in their life (question U2a in Appendix 1). Participants were queried for pathogens and infectious conditions, socioeconomic and demographic status, drug use and lifestyle variables (Table 1). The timing of depression and infection was not noted in the directory. Our results therefore correlate depression with any of these variables without reference to the order of occurrence. Demographic details are presented in Table 2.

**Table 1.** Information gathered from the registry

Category of information	Information recorded
Demographic and socioeconomic	Age, race, marital status, level of education, annual household income, urban/rural residence
Lifestyle variables	Use of tobacco, alcohol, marijuana, over-the-counter and prescription medications, illegal drugs
Gynecological health	*Chlamydia trachomatis*, *Neisseria gonorrhoeae*, *Treponema pallidum*, *Trichomonas vaginalis*, HIV, HPV, human herpes simplex 2, ‘frequent yeast infections’, ‘frequent vaginal infections’, pelvic inflammatory disease, premenstrual syndrome/premenstrual dysphoric disorder

**Table 2.** Descriptive characteristics of the study population

Demographic characteristics	*N* (%)
*Age*	
18–24	212 (14%)
25–34	822 (54.4%)
35–40	476 (31.5%)
*Race*	
White	1361 (91.3%)
Other	130 (8.7%)
*Marital status*	
Married	859 (57.2%)
Separated	45 (3.0%)
Divorced	127 (8.5%)
Never married	463 (30.8%)
*Education*	
Some college, vocational/technical	320 (21.2%)
Bachelor's degree	409 (27.1%)
Postgraduate	445 (29.5%)
*Urban/rural*	
Urban	982 (65.3%)
Rural	521 (34.7%)
*Appalachian*	
Yes	334 (22.2%)
No	1169 (77.8%)

Immune suppression and state of infection often vary with the menstrual cycle (Doyle *et al.* 2007). Subjects were therefore excluded from the study if they reported menstrual disruption or cessation, were using hormonal birth control or hormone replacement therapy, were pregnant or postpartum, or had undergone an ovarectomy or hysterectomy (the last two were not distinguished in the registry).

For bivariate comparisons, chi-squared tests were performed. Boneferroni corrections were used to account for multiple comparisons. Although the correlations between the individual variables and reports of repression provide useful descriptive information, the possibility for correlations among the included pathogens, individual demographics and risk behavior indicator variables necessitate a multivariate analysis. For a multiple regression analysis we estimated the probability of an individual reporting depression of at least two weeks duration using a linear probability model (LPM) in which depression was considered as a function of participant characteristics and infection variables. In this analysis we used three separate models, focusing first on infection variables alone, then adding sequentially demographic and behavioral characteristics to better isolate the independent relationships between specific pathogens and depression.

The models estimating the probability of an individual reporting depression and the explanatory variables can be written as follows:1
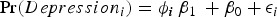
2
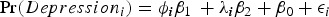
3
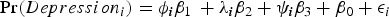
where ***ϕ***_***i***_ represents a vector of binary indicators for whether individual *i* reported experiencing each of the infection variables (Table 4 rows 1–6), ***λ***_***i***_ is a vector of indicators for participant characteristics of individual *i* (Table 4 rows 7–18) and ***ψ***_***i***_ is a vector of binary indicators for risk behaviors (Table 4, rows 19–29). Unique coefficients for each of the infections *β*_1_, each demographic characteristic *β*_2_ and each risk behavior *β*_3_ are reported in Table 4. The *β*_0_ represents the intercept, and *ε*_*i*_ represents a heteroskedasticity-robust individual error term. Analyses were conducted using the standard *Stata* packages logit, probit and regress for multiple logistic regression, probit regression and linear probability models, respectively.

This study was approved by the Institutional Review Boards at Bellarmine University (approval number 0313-3) and the University of Kentucky.

## Results

### Depression

A total of 1510 women met the inclusion criteria and provided information on mental health; 74% of them reported depression. Bivariate tests showed statistically significant positive associations of depression with *Chlamydia trachomatis*, *Trichomonas vaginalis*, abnormal pap smears, HPV, herpes simplex infections, endometriosis, infertility, frequent yeast infections and unspecified vaginal infections (Table 3). The sample size was sufficient to detect a statistically significant difference (at *p* < 0.002 for a 2% difference in positivity) for each pathogen except for HIV and *T. pallidum*, for which there were only one and zero positive subjects, respectively.

**Table 3.** Bivariate associations between depression and sexually transmitted pathogens

	Depression		
Agent or condition	Present	Absent	*p*-Value
*Chlamydia trachomatis*	80 (8.2%)	21 (4.0%)	0.002
*Neisseria gonorrhoeae*	14 (1.4%)	4 (0.8%)	0.250
HPV	146 (15.1%)	54 (10.4%)	0.01
*Candida albicans*	78 (8.0%)	22 (4.2%)	0.004
*Trichomonas vaginalis*	37 (3.8%)	2 (0.4%)	<0.0001
Herpes simplex virus	45 (4.6%)	16 (3.1%)	0.14
Pelvic Inflammatory Disease	18 (1.9%)	5 (1.0%)	0.180
Frequent vaginal infections	40 (4.1%)	6 (1.13%)	0.0014

Note: Numbers refer to the total number of subjects that tested positive for the pathogen. Percentages in parentheses refer to the percentage of all subjects in the category that were positive for the specified pathogen. Too few reported positivity for *T. pallidum* and HIV to meet minimum sample sizes for statistical testing.

**Figure 1.**
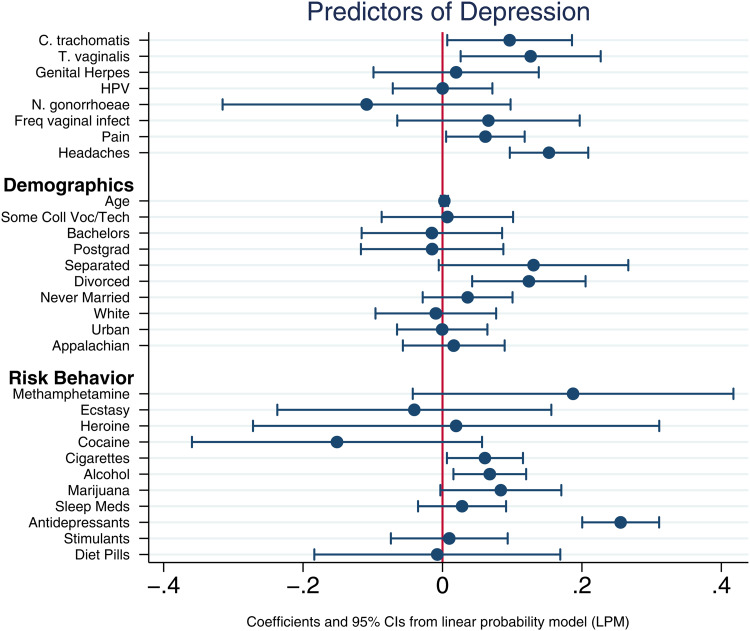
Linear probability model predicting reports of depression. Points represent coefficients from the most inclusive linear probability model (Table 4, column 3). Bars represent 95% confidence intervals around each point. The first eight rows correspond to infection variables and two variables (pain and headaches) for which infection may be a contributing factor (Doyle *et al.*, 2015).

The only pathogens that were significantly correlated with depression in the multiple regression analysis were *C. trachomatis* and *T. vaginalis*. Among the demographic variables, the only significant correlates of depression were use of antidepressants, being divorced and smoking cigarettes or marijuana (Figure 1). Results are robust to alternative model specifications and standard logistic diagnostic tests for omission of high-leverage, standardized residuals and deviance.

We report results from heteroskedastic robust LPM for ease of interpretation, although *p*-values and other patterns are consistent across alternative modeling strategies, including probit and logistic regression models (Appendices 2 and 3, respectively).

Table 4 presents the results of the three LPM models indicating that the inclusion of more covariates highlights specific pathogens as substantively important predictors of reported depression. While several reported infection variables (HPV, frequent vaginal infections, *C. trachomatis* and *T. vaginalis*) are significant predictors in the model that account only for other infections (Table 4, column 1), the only infection variables that were significantly correlated with depression in the multiple regression analyses that account for the full set of demographic and behavioral covariates (Table 4, columns 2 and 3) are *C. trachomatis* and *T. vaginalis*, with magnitudes similar to those for headaches and pain. All else equal, each of these pathogens is associated with roughly a 10-percentage point increase (0.09 and 0.12, respectively) in the likelihood of reporting depression. Among the demographic and risk behavior variables, the only significant correlates of depression are use of antidepressants, alcohol, being divorced and smoking cigarettes. Associations of separation and marijuana usage are suggestive (*p* < 0.10) but not statistically significant. Figure 1 visually depicts the results of the most inclusive model (Table 4 column 3).

**Table 4.** Linear probability models for variables associated with depression

	(1)	(2)	(3)
*C. trachomatis*	0.098*	0.089*	0.096*
	[0.009–0.186]	[0.005–0.173]	[0.007–0.186]
*Trichomonas vaginalis*	0.240**	0.173**	0.127*
	[0.153–0.327]	[0.084–0.263]	[0.026–0.227]
Genital herpes	0.046	0.045	0.020
	[−0.069–0.160]	[−0.076–0.167]	[−0.099–0.138]
HPV	0.068*	0.038	0.000
	[0.000–0.136]	[−0.032–0.108]	[−0.071–0.072]
*Neisseria gonorrhoea*	−0.010	−0.089	−0.109
	[−0.203–0.183]	[−0.278–0.100]	[−0.315–0.098]
Frequent vaginal infections	0.131*	0.109†	0.066
	[0.014–0.248]	[−0.016–0.234]	[−0.065–0.197]
Pain		0.137**	0.062*
		[0.086–0.188]	[0.005–0.118]
Headaches		0.153**	0.153**
		[0.096–0.210]	[0.097–0.209]
Age		0.004	0.003
		[−0.001–0.009]	[−0.003–0.008]
Some college, vocational or technical		0.023	0.007
		[−0.064–0.111]	[−0.087–0.101]
Bachelors		0.005	−0.015
		[−0.087–0.098]	[−0.116–0.086]
Postgraduate		−0.013	−0.015
		[−0.107–0.081]	[−0.117–0.087]
Separated		0.188**	0.131†
		[0.070–0.306]	[−0.005–0.267]
Divorced		0.140**	0.124**
		[0.059–0.220]	[0.043–0.205]
Never married		0.048	0.036
		[−0.014–0.111]	[−0.028–0.100]
White		0.027	−0.010
		[−0.058–0.112]	[−0.096–0.077]
Urban		0.008	−0.000
		[−0.056–0.071]	[−0.065–0.064]
Appalachian		0.026	0.016
		[−0.046–0.098]	[−0.057–0.089]
Methamphetamine			0.187
			[−0.043–0.417]
Ecstasy			−0.040
			[−0.237–0.156]
Heroin			0.020
			[−0.272–0.311]
Cocaine			−0.151
			[−0.359–0.057]
Cigarettes			0.061*
			[0.006–0.115]
Alcohol			0.068*
			[0.016–0.120]
Marijuana			0.084†
			[−0.003–0.170]
Sleep medications			0.028
			[−0.035–0.091]
Antidepressants			0.256**
			[0.200–0.311]
Stimulants			0.010
			[−0.074–0.094]
Diet pills			−0.007
			[−0.184–0.169]
*R*^2^	0.02	0.08	0.14
*N*	1479	1430	1363

Note: 95% confidence intervals based on Huber–White robust standard errors in brackets below coefficients from three separate LPMs. In column 1 depression is regressed on the six infection variables. Column 2 adds controls for headaches, pain, and demographic characteristics. Column 3 adds controls for risk behavior indicators. †*p* < 0.1; *p < 0.05; **p < 0.01.

The previous study investigating associations between depression and sexually transmitted pathogens (Doyle *et al.* 2015) reported a significant association of depression with *C. trachomatis* and a non-significant association with *T. vaginalis*. Combined probability tests evaluating the overall findings of that study and the present one using the same (i.e. logistic) statistical model revealed a statistically significant association for *C. trachomatis* (*p* < 0.001, *χ*^2^ = 22.87, d.f. = 4) and *T. vaginalis* (*p* < 0.05, *χ*^2^ = 9.79, d.f. = 4), but not for any of the other pathogens. Depression occurred more frequently in women who reported infections with *C. trachomatis* and *T. vaginalis* (89.5%) than with either *C. trachomatis* (57.5%) or *T. vaginalis* (45.8%) alone (*p* < 0.025, *χ*^2^ = 11.62, d.f. = 4).

## Discussion

Our results show that several sexually transmitted pathogens were significantly associated with depression by bivariate analyses; however, only two of the tested pathogens – *Chlamydia trachomatis* and *Trichomonas vaginalis* – were significantly associated with depression after correlations among infections and pathogens were accounted for by multiple regression analysis. *C. trachomatis, T. vaginalis* and the pathogens that were not significantly associated with depression generate inflammation (Georgescu *et al.* 2018; Hafner *et al.* 2014; Hube *et al.* 2015; Stevens and Criss 2018; Thurman and Doncel 2011). The results therefore support the hypothesis that depression results from specific effects of particular pathogens rather than from a general inflammatory response associated with infection.

The lack of association between *N. gonorrhoeae* and depression is noteworthy because the pathologies of *N. gonorrhoeae* and *C. trachomatis* are similar. Each species can cause purulent discharges, pelvic inflammatory disease, oviduct inflammation and scarring, infertility and ectopic pregnancy (Hafner *et al.* 2014; Stevens and Criss 2018). If the depression associated with *C. trachomatis* resulted from the awareness of the presence of these manifestations, then *N. gonorrhoeae* should have been similarly associated with depression. This difference also bears on the lack of information in the database concerning the timing of knowledge about *C. trachomatis* infection relative to the timing of depression by weakening the possibility that its association with depression resulted from an effect of knowledge about a urethritis-associated sexually transmitted disease.

The inflammation associated with *C. trachomatis* and *N. gonorrhoeae* involves elevation of interferon gamma and activity of neutrophils (Hafner *et al.* 2014; Stevens and Criss 2018). Interferon gamma induces expression of indolamine 2,3-dioxygenase, which degrades tryptophan and thereby contributes to tryptophan restriction (Chen 2011; Ziklo *et al.* 2018). The difference between *N. gonorrheae* and *C. trachomatis* with respect to depression therefore cannot be attributed to the absence of this trigger for tryptophan restriction. Correlations between cytokines and depression are, however, complex, defying simple cause/effect explanations (Geisler *et al.* 2018). IDO1 and hence tryptophan can be controlled by different mechanisms, and *C. trachomatis* can elevate IDO1 independently of interferon gamma (Ziklo *et al.* 2019).

Upon tryptophan restriction *in vitro*, genital serovars of *C. trachomatis* enter a quiescent persistent phase (Aiyar *et al.* 2014). When indole is present, the tryptophan synthase of *C. trachomatis* generates tryptophan from the indole, allowing *C. trachomatis* to emerge from this quiescent phase to multiply and spread in the presence of tryptophan restriction (Aiyar *et al.* 2014). Inflammation associated with *N. gonorrhoeae* is not known to be associated with resistance to tryptophan restriction. The ability of *C. trachomatis* to persist in the presence of tryptophan restriction accords with the possibility that tryptophan restriction may be less effective in controlling *C. trachomatis* than *N. gonorrhoeae* and thus *C. trachomatis* may be associated with more persistent tryptophan restriction and, consequently, depression.

The hypothesized mechanism for an effect of *C. trachomatis* on depression presumes that tryptophan restriction at the sites of infection lowers tryptophan levels in the blood sufficiently to reduce serotonin synthesis in the brain. Lowered plasma concentrations of tryptophan are thought to reduce tryptophan in the brain as a result of the competition between amino acids at the blood–brain barrier (Fernstrom and Wurtman 1997; Pardridge 1979; Schiepers *et al.* 2005). As a result of this competition, even a small reduction in systemic tryptophan might lower serotonin synthesis in the brain (Fernstrom and Wurtman 1997). The greater the tryptophan sink is, however, the greater the potential for a reduction in serotonin synthesis. Serum concentrations of tryptophan average about 25% lower in depressed subjects than in subjects with normal mood (Cowen *et al.* 1989).

Persistent, systemic *C. trachomatis* infections should tend to generate a more substantial tryptophan sink and thus lower mood than infections that are restricted to the urogenital tissue. Reiter's syndrome is the main recognized category of systemic *C. trachomatis* disease. It is a persistent autoimmune disease that encompasses reactive arthritis and uveitis, with *C. trachomatis* being found in the joints and the conjunctiva of the eye, respectively (Haller-Schober and El-Shabrawi 2002; Rihl *et al.* 2006). *C. trachomatis* reaches the joints via infected monocytes or macrophages (Rihl *et al.* 2006). Infection of the eye occurs mainly through autoinoculation by urogenitally contaminated hands.

Integration of genetic associations of Reiter's syndrome provides a more comprehensive framework for evaluating the contribution of *C. trachomatis* to depression. The HLA-B27 allele is present in about 75% of subjects with sexually acquired Reiter's syndrome, which is caused mostly by *C. trachomatis*; the allele is present in about 90% of individuals with chronic disease (Baguley and Greenhouse 2003). Pathological effects of HLA-B27 appear to occur in response to microbes: HLA-B27 transgenic rats have inflammatory joint disease except when they are germ-free (Taurog *et al.* 1994). Depression has been found to be more common in uveitis patients who were HLA-B27 positive, with about half of these subjects being mildly or clinically depressed according to Beck Depression Inventory tests (scores ≥ 9; Maca *et al.* 2011). These findings accord with the hypothesis that persistent, systemic *C. trachomatis* infections may particularly associated with depression. We expect that *C. trachomatis*-infected reactive arthritis patients would also be prone to depression, but to our knowledge this possibility has not been investigated.

The mechanistic reason for the association between *T. vaginalis* and depression is unclear, but *T. vaginalis* synthesizes indole which may be used by *C. trachomatis* as a substrate for synthesis of tryptophan (Aiyar *et al.* 2014; Lloyd *et al.*n 1991; Zubacova *et al.* 2011) and thus foster persistence of *C. trachomatis* (Ziklo *et al.* 2016). The higher frequency of depression among women who reported having both *C. trachomatis* and *T. vaginalis* infections is consistent with an exacerbating effect of *T. vaginalis* on *C. trachomatis*.

Our test focused on sexually transmitted pathogens because they tend to be persistent, but the arguments should apply to other pathogens that can persist in the presence of tryptophan restriction as a result of an ability to synthesize their own tryptophan. The presence of tryptophan synthase is not necessarily an indicator of this ability – *C. trachomatis* is functional in the presence of tryptophan restriction by virtue of mutations in its tryptophan synthase operon (Somboonna *et al.* 2019), suggesting an evolutionary arms race between host abilities to silence tryptophan synthesis and the ability of *C. trachomatis* to circumvent this silencing. To our knowledge the only other pathogen for which tryptophan synthesis is known to be used as part of a persistence strategy is *Mycobacterium tuberculosis*, which upregulates tryptophan synthase upon infection of epithelial cells (Ryndak *et al.* 2015) and uses tryptophan-embedded phagosomal membranes to prevent fusion of the phagosome with lytic vesicles in macrophages, thereby evading intracellular destruction (Ferrari *et al.* 1999; Meena and Rajni 2010). Deletion of a tryptophan synthase gene in *M. tuberculosis* reduced persistence within macrophages *in vitro* as well as in lungs and spleen in a murine model (Smith *et al.* 2001). As is the case with *C. trachomatis*, failure of tryptophan restriction to resolve *M. tuberculosis* infection may therefore lead to persistent tryptophan restriction and low mood. Accordingly, depression is a major manifestation of tuberculosis (Ige and Lasebikan 2011).

The recognition that particular pathogens are associated with depression bears on the interpretation of drug effects. Minocycline, for example, ameliorates depression, an association that has been interpreted to result from its direct anti-inflammatory effects (Rosenblat and McIntyre 2018). Minocycline is effective against *C. trachomatis* (Romanowski *et al.* 1993) and *M. tuberculosis* (Deshpande *et al.* 2019) and could therefore ameliorate depression by controlling these pathogens. Our results therefore emphasize the need to evaluate the extent to which any ameliorative effects of antimicrobials are due to suppression of pathogens relative to direct suppression of inflammation.

We think that the main value of our findings lies in indicating directions for future study. The correlational nature of our data do not allow us to assign cause and effect. Knowledge about the presence of *C. trachomatis* infection might have contributed to depression, even though the lack of an association of depression with *N. gonorrhoeae* indicates that knowledge about the presence of a *C. trachomatis* infection is insufficient to generate the observed association. The lack of adequate sample size for HIV and *T. pallidum* does not allow us to evaluate an association of these pathogens with depression. Each has been associated with depression but the psychological effects of knowledge of chronic syphilis or AIDS on mood has not been distinguished from direct effects of the pathogens on mood.

The absence of information on temporal sequence of depression and other variables in our database prevents an assessment of whether infection occurred before depression or vice versa. Women could have an infection without knowing it because infections are often asymptomatic (e.g. with *N. gonorrhoeae* and *C. trachomatis*) and may have erred in reporting depression or infectious conditions. These factors undoubtedly will create variability in the results but should not have generated the observed associations. The presence of an association of depression with *C. trachomatis* but the absence of such an association with *N. gonorrhoeae* serves as a control for these uncertainties, because the symptoms of these infections and the prevalence of asymptomatic relative to symptomatic infections are similar for these two pathogens. We note, however, that numbers of infected individuals were still relatively small, particularly for *N. gonorrhoeae* and *T. vaginalis*, a fact that could influence the outcome of statistical testing. Additional analyses with larger samples sizes and assessment of the temporal sequence of infection relative to depression will be useful.

Animal studies may provide a useful direction for evaluating cause and effect as well as the potential for generality of infectious causation of depression beyond humans. One limitation of animal models is the difficulty in knowing whether an animal is depressed. Social withdrawal could result from other phenomena, such as malaise, pain, distrust, fear or insecurity. One of the most reliable examples of infection-induced depression in another host species is sad horse disease, which is caused by borna disease virus and is associated with affect that corresponds to human depression (Tizard *et al.* 2016). In rats, borna disease virus infections of the central nervous system are associated with elevated IDO, suggesting that they also probably produce reductions in tryptophan (Formisano *et al.* 2017).

Animal models may also be helpful for evaluating alternative mechanisms. Mice infected with Bacille Calmette Guérin (= BCG, a strain derived from *M. tuberculosis bovis*) appear chronically depressed. BCG induced cytokines (TNF alpha and interferon gamma), which led to tryptophan catabolism via IDO. Accordingly, pre-treatment with an IDO inhibitor blocked BCG-induced depression, and IDO-deficient mice were resistant to BCG induction of depression even though they produced inflammatory cytokines associated with BCD infection (Moreau *et al.* 2005; O'Connor *et al.* 2009). These findings support a contribution of IDO activity to depression that is distinct from other effects of inflammatory cytokines.

Our results suggest that research needs to look beyond inflammation *per se* to processes associated with specific aspects of the inflammatory response, such as tryptophan restriction, and the effects of particular pathogens. The set of pathogens considered in this study was limited to those reported to the health registry. Our data set did not include, for example, cytomegalovirus infection, which causes persistent infections and has been correlated with depression in studies that did not find associations between depression and general markers of inflammation (Simanek *et al.* 2014; Simanek *et al.* 2018a, b). Cytomegalovirus has been associated with tryptophan degradation *in vivo* (Sadeghi *et al.* 2012). Similarly, *H. pylori*, which has been associated with depression in women (Simanek *et al.* 2018a), has also been associated with elevated levels of the tryptophan-degrading enzyme, IDO1 (Larussa *et al.* 2015). Each of these two pathogens can cause infections that may persist for decades and may therefore be associated with chronic lowering of tryptophan levels resulting in depressed mood.

The associations of particular persistent infectious agents (*C. trachomatis*, *M. tuberculosis*, *H. pylori* and cytomegalovirus) with depression, tryptophan depletion and resistance to tryptophan depletion support the hypothesis that at least some depression may result from evolutionary arms races with particular pathogens. The presence of tryptophan restriction by IDO1 in rodents suggests a deep evolutionary presence of this adaptation in placental mammals. More broadly, molecular phylogenies indicate that IDO1 and its high affinity for tryptophan occurs in monotremes, marsupials and placentals, but not in birds, amphibians or fish. This pattern suggests that IDO1 evolved its tryptophan catabolic functions in monotremes from an IDO2-like molecule (Yuasa *et al.* 2007; Yuasa *et al*. 2009). This pattern suggests that tryptophan restriction has evolved as a mammalian adaptation for regulating tryptophan and potentially as defense against pathogens before the divergence of these three groups of mammals. A short-term depressive effect on mood may have been a component of this defense by encouraging rest and recovery. Selection on pathogens to persist, however, apparently has led to persistent tryptophan restriction as an ineffective by-product of the adaptive response that is ineffective for the particular pathogens that evolved the upper hand in the evolutionary arms race associated with this defense. This interpretation together with the findings reported in this paper draw attention to the need for research on infection, inflammation and depression that encompasses the entire spectrum of persistent infectious agents with attention to the possible effects that particular pathogens have on depression through induction of persistent tryptophan restriction.

## Data Availability

The data that support the findings of this study are available from The University of Kentucky's Wellness, Health and You data resource (https://www.wellnesshealthandyou.org), formerly the Kentucky Women's Health Registry. Restrictions apply to the availability of these data. A data use agreement was signed to allow use by the authors and thus are not publicly available from the authors.

## Appendix 1: Questions from the Kentucky Women's Health Registry survey version 8.1 related to depression and infection

**Table:**

Registry question	Optional answers
C2. Your health:	Allows full activity **– Skip to C3** Limits your activities **– Skip to C2a** Chose not to answer**– Skip to C3**
C2a. The problem that limits your activity is: CHOOSE ALL THAT APPLY	Pain in your muscles or joints Fatigue, tiredness, or lack of energy Shortness of breath or difficulty breathing Heart problems including chest pain Depression, feeling blue, or nerve problems None of these Chose not to answer
U2. Has there been a period of at least two straight weeks when you have: CHOOSE ALL THAT APPLY	In your In the past Chose not lifetime 12 months No to answer Felt down, depressed or hopeless? □ □ □ □ Had little interest or pleasure in doing things? □ □ □ □ Had trouble concentrating on things? □ □ □ □ Had panic or episodes or panic attacks? □ □ □ □
E9. Have you ever had any of the following sexually transmitted diseases? CHOOSE ALL THAT APPLY	Genital herpes Human papilloma virus (HPV) or genital warts Pelvic inflammatory disease Gonorrhea *Chlamydia* Syphilis *Trichomonas* Human immunodeficiency virus (HIV) or AIDS Other:________________________________________________ None Chose not to answer

## Appendix 2: Probit estimates of association with depression

**Table:**

	Probit 1	Probit 2	Probit 3
*Chlamydia trachomatis*	0.329*	0.347*	0.416*
	(0.151)	(0.165)	(0.202)
*Trichomonas vaginalis*	1.135**	1.007**	0.792*
	(0.346)	(0.348)	(0.370)
Genital herpes	0.129	0.105	0.022
	(0.183)	(0.202)	(0.222)
HPV	0.195†	0.119	0.003
	(0.104)	(0.109)	(0.121)
*Neisseria gonorrhoeae*	0.056	−0.252	−0.348
	(0.349)	(0.338)	(0.356)
Frequent vaginal infections	0.515*	0.418	0.275
	(0.253)	(0.271)	(0.285)
Pain		0.384**	0.161*
		(0.072)	(0.080)
Headaches		0.413**	0.438**
		(0.078)	(0.082)
Age		0.010	0.007
		(0.008)	(0.008)
Some college, vocational/technical		0.082	0.055
		(0.136)	(0.155)
Bachelors		0.027	−0.011
		(0.139)	(0.162)
Postgraduate		−0.026	−0.009
		(0.140)	(0.163)
Separated		0.669*	0.495
		(0.276)	(0.304)
Divorced		0.455**	0.464**
		(0.149)	(0.163)
Never married		0.128	0.095
		(0.091)	(0.098)
White		0.086	−0.022
		(0.127)	(0.133)
Urban		0.027	0.025
		(0.094)	(0.100)
Appalachian		0.073	0.062
		(0.107)	(0.113)
Ecstasy			0.017
			(0.584)
Heroin			−0.264
			(0.735)
Cocaine			−0.582
			(0.386)
Cigarettes			0.184*
			(0.087)
Alcohol			0.186*
			(0.080)
Marijuana			0.286†
			(0.164)
Sleep medicines			0.132
			(0.113)
Antidepressants			0.933**
			(0.123)
Stimulants			0.099
			(0.173)
Diet pills			−0.005
			(0.310)
*N*	1479	1430	1353
			

Note. Models mirror those described in Table 4 in the body of the text. Huber–White robust standard errors in parentheses. Coefficients from probit model. †*p* < 0.1; **p* < 0.05; ***p* < 0.01

## Appendix 3: logit estimates of association with depression

**Table:**

	Logit 1	Logit 2	Logit 3
*Chlamydia trachomatis*	1.715*	1.776*	2.107*
	(2.08)	(2.02)	(2.10)
*Trichomonas vaginalis*	7.846**	5.979*	3.847†
	(2.81)	(2.46)	(1.86)
Genital herpes	1.250	1.236	1.058
	(0.72)	(0.61)	(0.15)
HPV	1.387†	1.222	1.000
	(1.89)	(1.09)	(0.00)
*Neisseria gonorrhoeae*	1.066	0.616	0.524
	(0.11)	(0.89)	(1.12)
Frequent vaginal infections	2.444†	2.145	1.601
	(1.94)	(1.53)	(0.92)
Pain		1.871**	1.288†
		(5.31)	(1.91)
Headaches		1.956**	2.061**
		(5.29)	(5.30)
Age		1.017	1.011
		(1.37)	(0.84)
Some college, vocational/technical		1.135	1.063
		(0.56)	(0.24)
Bachelors		1.040	0.958
		(0.17)	(0.16)
Postgraduate		0.964	0.948
		(0.16)	(0.19)
Separated		3.295*	2.409
		(2.30)	(1.57)
Divorced		2.203**	2.172**
		(3.01)	(2.70)
Never married		1.251	1.146
		(1.47)	(0.83)
White		1.156	0.974
		(0.69)	(0.12)
Urban		1.035	1.038
		(0.23)	(0.22)
Appalachian		1.125	1.106
		(0.67)	(0.53)
Ecstasy			0.963
			(0.04)
Heroin			0.561
			(0.43)
Cocaine			0.385
			(1.37)
Cigarettes			1.367*
			(2.16)
Alcohol			1.364*
			(2.34)
Marijuana			1.754*
			(1.97)
Sleep medicines			1.268
			(1.23)
Antidepressants			5.179**
			(7.34)
Stimulants			1.196
			(0.60)
Diet pills			1.029
			(0.05)
*N*	1479	1430	1353

Note. Models mirror those described in Table 4 in the body of the text. Huber–White robust standard errors in parentheses. Coefficients from logistic regressions presented as odds ratios. †*p* < 0.1; **p* < 0.05; ***p* < 0.01.
